# Connected model to optimize performance

**DOI:** 10.3389/fspor.2022.1054783

**Published:** 2023-01-13

**Authors:** Lucie Lerebourg, Jérémy Coquart

**Affiliations:** ^1^ Univ. Rouen-Normandie, Laboratoire Centre D’Études des Transformations des Activités Physiques et Sportives (CETAPS - UR 3832), Mont-Saint-Aignan, France; ^2^ Univ. Lille, Univ. Artois, Univ. Littoral Côte D'Opale, ULR 7369 - URePSSS - Unité de Recherche Pluridisciplinaire Sport Santé Société, Lille, France

**Keywords:** modelling, big data, artificial intelligence, connected technologies, sport, run, estimation

## Introduction

**Figure 1:**
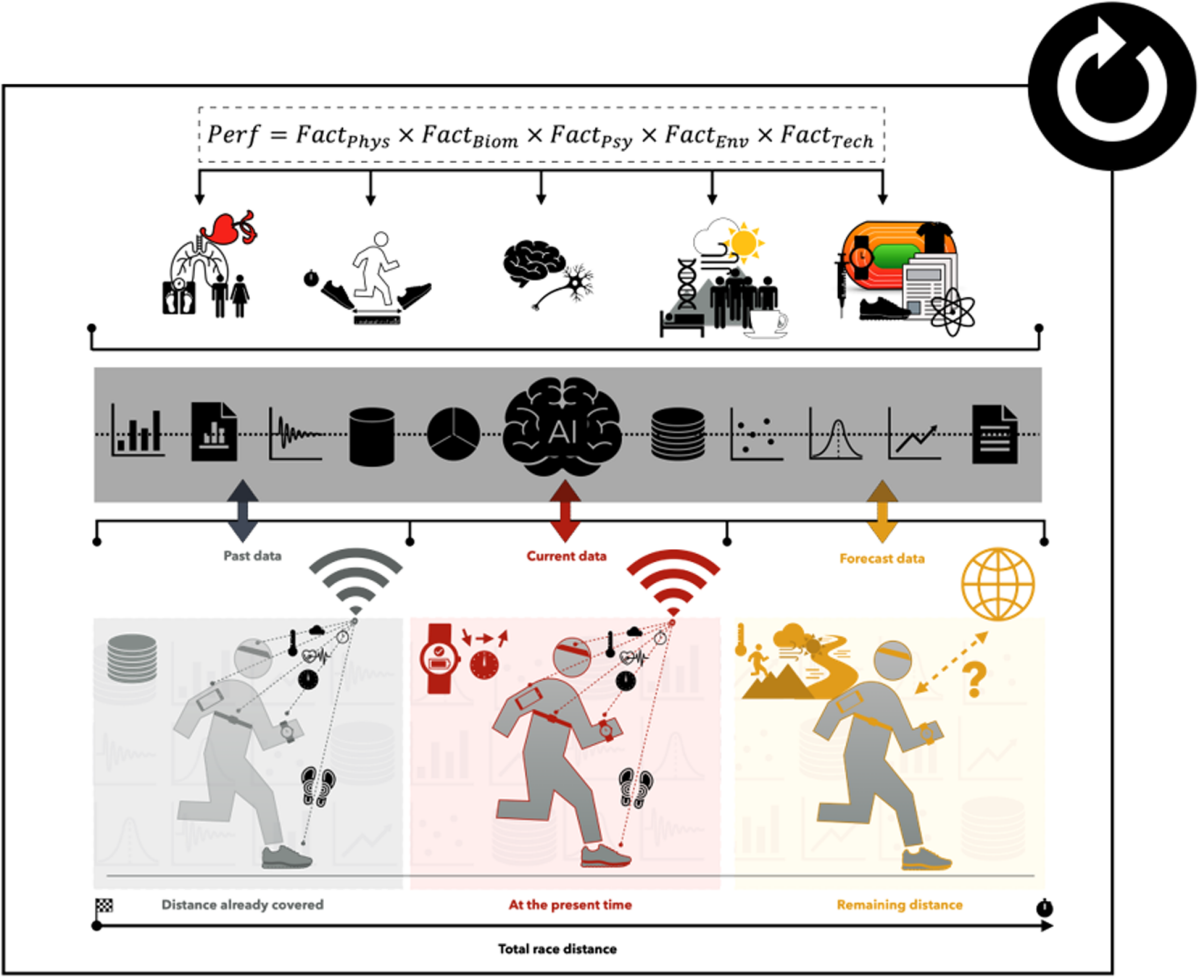
Illustration of a “connected multifactorial” intelligent model capable of adapting in “real” time during the effort to optimize running performance. $Fact_{Phys}$ (physiological factor), $\;Fact_{Biom}$ (biomechanical factor), $Fact_{Psy}$ (psychological factor), $Fact_{Env}$ (environmental factor), $Fact_{Tech}$ (technological factor), AI (artificial intelligence).

The analysis and prediction of running performance have been the subject of much research. Several tools using the relationship between distance (or speed) and its time limit, as well as physiological models were developed to understand human endurance and to explain performance based on physiological parameters (1–3). Tables (4), mathematical equations (e.g., logarithmic, hyperbolic, exponential, linear…) (5–7) including the concepts of critical speed (8, 9) or power law (3, 10), nomograms (11, 12), and Artificial Intelligence (AI) algorithms (13, 14) are notable examples. Although many approaches are valid and accurate to predict performance over a given distance (e.g., nomograms, concept of critical velocity or power law…) (15), these approaches notably allow the prediction of a “final time”, which could be commonly referred to as performance. It is not uncommon for the running performance predictions are based on the theoretical calculation of running time using the best performance(s) achieved over other distances, and on some equivalence between the time references of the different distances covered (12, 15–17). However, prediction approaches, more and more elaborate considering empirical, biomechanical or physiological data, have been developed over the years (18, 19), notably through the evolution of technologies such as AI (14, 19, 20). These prediction approaches can be useful for calibrating, quantifying sessions, but also detecting, for example, future athletes with high potential (16, 17, 19, 21). They can also provide additional information (e.g., identify specific training intensities…) (16, 17, 21) to traditional laboratory methods measuring the main physiological parameters of running performance (e.g., maximal oxygen uptake: $\dot{V}O_{2\max}$, maximum aerobic speed, aerobic endurance capacity, etc.) (8, 22). However, beyond the predicted time, it could be interesting to question the conditions for achieving this final time, in other words, to question the “path” that the athlete should take to reach it. Indeed, if performance prediction can be useful to optimize performance, to define specific training intensities, to plan split times during competitions (16, 17, 21), this does not necessarily mean that the average speed obtained through the predicted final time, to achieve performance, must be constant throughout the distance covered. The approaching condition of running could then be rethought other than by the fact that a constant or regular speed is ideal by focusing in particular on other physiological parameters than $\dot{V}O_{2\max}$, the energy cost and the endurance capacity commonly used in performance modeling [i.e., paradigm of constant speed from the Di Prampero equation (23)]. To achieve a performance, we could for example ask ourselves about the optimal speed (e.g., target speed) and the strategy for managing it (e.g., constant speed, pace variation…) but also the conditions for achieving it (e.g., weather conditions, race profile, diet, sleep, sports equipment, technologies…), which can delay voluntary exhaustion, but also allow running the given distance as quickly as possible (24–31). To contextualise this, we can take the example of the “Ineos 1:59 Challenge” project, where Kenyan Eliud Kipchoge aimed to break the iconic 2-h barrier in the marathon. The result is that strategies such as a relatively “regular” running pace (i.e., 2 min 49 s per km) as well as the use of “new generation” running shoes (e.g., shoes with carbon plates and rubber) have proven to be effective, It should be noted that a similar event took place in the United States in the early 1990s, where the runners’ performance was not homologated by World Athletics for various reasons (e.g., intermittent pacers, car emitting a laser beam…). From these observations, we can then be led to wonder beyond the final time that could be predicted, about the “ideal” running pattern that could optimise running performance while taking into consideration (e.g., in real time from connected objects…) the multifactorial aspect of the latter (i.e., physiological, biomechanical, psychological, environmental and technological factors) (cf. Figure 1) (24–31). Therefore, the aim of this opinion is to try to answer these questions. For this, we consider turning to Big Data (i.e., megadata collected to designate a set of digital data produced by the use of new technologies) and AI, which seem to offer new work perspectives for the prediction of sports performance (13, 20, 32–36).

## The current landscape

### Big data and connected technologies

At present, increasing amounts of data are collected in many disciplines including running, in particular through sensors (e.g., Global Positioning Sensor: GPS, accelerometer, heart rate monitor…), connected objects (e.g., smart meters such as watches, glasses, textiles, insoles…) (37–39) content published on databases (e.g., performance, split times during competitions, results…). If we are interested, for example, at these data commonly collected in running *via* connected devices and/or smartphone applications (e.g., Strava®, Garmin®, Runtastic®…) (40), the latter can make it possible to analyze, or even predict, performance by monitoring several variables (e.g., pace or speed of movement, variation in the altitude difference of the course, amplitude and frequency of strides…) in a non-invasive way in real conditions (i.e., with possible real-time feedback) and especially outside a laboratory (14, 34, 37, 41–43). The research of Emig and Peltonen (34), Smyth and Muniz-Pumares (30) has notably highlighted mathematical modelling based on the use of connected wearable technologies such as wrist devices (e.g., connected watches) or smartphones to correlate performance indices with the volume and intensity of training in order to quantify, for example, the optimal training load. These studies (30, 34) have chosen to integrate in their algorithms (already taking into consideration past references on the target distance), the runners’ training regime (e.g., distance, time, running pace and elevation gain) 6 weeks before the prepared competition. The use of these connected technologies and Big Data, suggests new ways of quantifying and predicting athletic performance in real conditions. Other technologies, still not widely used in the running world, such as connected insoles could also allow the collection and exploitation of new data in order to understand and optimize sports performance (e.g., integration of these data in prediction algorithms) (44, 45). However, beyond the quality of the recovered data (i.e., precision, accuracy with over or underestimation of raw values, like recorded energy expenditure depending running intensities, but also distances significantly underestimated and less accurate in the forest areas to the road area, that can highlight the limitations of connected objects) (46–48), the exploitation of these data could be complex in view of the quantity of available data (Big Data) obtained through connected technologies. What methodology/approach could then respond to this Big Data problem?

### Artificial intelligence

AI used in many fields of science (e.g., meteorology, medicine, sport sciences…) (32, 49, 50) suggests new ways of quantifying and predicting sports performance in real-life conditions, given the scientific publications obtained in recent years. Indeed, Hammerling et al. (20) performed predictions of race times in the 2013 Boston Marathon for all runners who reached the halfway point of the race but did not have the opportunity to cross the line due to an attack (i.e., interruption of the race following the explosion of two bombs placed near the finish line) and thus to recognize the achievements of these runners. To make these predictions, Hammerling et al. (20) used a database of all previous years’ performances at the same event (i.e., Boston Marathon of 2010 and 2011) and took into account the “real” times (i.e., time intervals of 5 km as well as the final 2,195 km) of the runners engaged in this event before they were interrupted by the organization. The use of different AI algorithms including K-Nearest Neighbors (KNN), made it possible to predict the performance of all the runners (i.e., a final time based on the prediction of intermediate times), i.e., the time they could have achieved over the distance, to establish a ranking of the runners involved in this event. To do this, authors created an independent validation dataset from a fraction of the runners (25.7%) who finished the race and then ran dropout simulations at various points of the race on these runners in the same proportion as the true runners who unfortunately did not finish. The predicted finish times of this sample of fake runners who drop out were then compared with the actual finish times of these runners to assess the effectiveness of the statistical approaches. These predictions shown to be relatively accurate (i.e., Mean Absolute Error of 1 min 30 s on average) with an increased accuracy for runners who had to abandon later. In addition, beyond the prediction of performance, *via* AI, based on real data recovered during competitions, other work (14) has highlighted the use of supervised learning algorithms based on real training conditions in amateur runners to predict marathon performance, for example.

### Modelling project

Given the multifactorial aspect of performance (25–31, 51) and the fact that performance prediction is a subject of great interest to athletes and coaches, we could “legitimately” ask ourselves what future prediction models might look like? Taking into account previous work in this field of AI and the current evolution of connected technologies such as textiles or insoles, would the challenge then be to think of an approach, an equation that is able to “simply” optimize a large number of factors correlated with past data (e.g., data based on training program or even the start of a race), “actual” (i.e., data obtained in real time, such as heart rate) and/or future (e.g., estimates based on future conditions and forecasts during a race, such as changes in weather conditions) of running performance by connected objects? Could we not try to propose a multifactor equation:

$$Perf=Fact_{Phys}\times Fact_{Biom}\times Fact_{Psy}\times Fact_{Env}\times Fact_{Tech}$$

where each physiological ($Fact_{Phys}$), biomechanical ($Fact_{Biom}$), psychological ($Fact_{Psy}$), environmental ($Fact_{Env}$) and/or technological ($Fact_{Tech}$) factor would be expressed through the intermediary and preponderance of indices conducive to performance without taking the risk of straying into “prediction-fiction”. In this case, it would be a matter of extracting and using data from wearable devices to identify potential performance indices and then transform them into significant parameters with the ultimate objective of designing a “fair” and “accurate” modeling of running performance. This would be an “intelligent” model capable of taking into account a large amount of information based on physiological factors (e.g., values of critical speed, HR, acceleration, muscle oxygenation, body temperature, hydration rate…), biomechanics (e.g., values of amplitude and frequency of the stride, strength, muscle power, foot placement on the ground…), psychological (e.g., stress indices, motivation, psychological state or personality trait related to the challenge of the competition), environmental (e.g., weather indices, course profile, context of the race…) and technological (e.g., energy storage/return values of shoes, aerodynamic values of textiles) in order to be able to “coach” the athlete at the present time (“T” time), either to indicate to him/her, for example, to accelerate, stabilize or reduce his/her running speed according to the effort he is making and the effort he/she will still have to make with the “ultimate” objective of optimizing sports performance (Figure 1). To develop such a formula, we could use, for example, multiple regression to extract and use the relevant data in the model. However, we should be careful about the risk of multicollinearity if one of the explanatory variables in a model is a combination of one or more other explanatory variables in that model, thus distorting the coefficient estimates.

## Discussion

The use of connected technologies combined with complex algorithmic methods, such as AI, could offer new perspectives for modeling and/or predicting running performance. However, performance modeling based exclusively on connected data as well as the use of an AI method due to a large amount of data could be relatively limited in relation to:

▪ The quantity and quality of raw data extracted. How to limit the performance prediction bias related to the precision and/or accuracy of connected devices (46–48)?
▪ The relevance of some data (i.e., parameters using to qualify or define performance according to existing inter-individual differences between runners or type of race, for example).
▪ The scientific mastery needed to make sense of the data (e.g., modeling procedure defining the algorithms) (52) and to obtain valid results with respects to the varieties of different algorithmic approaches that can be applied to the same data set (e.g., the ratio used for the data sets of the same size, the ratio used for training and test datasets, the number of hidden layers or the training rate for training a neural network, the number of k in KNN, the type of distance in KNN…) (14, 32, 53, 54)…

Thus, while this perspective of a “connected multifactorial” model seems to be “relatively simple” because data can easily be made public or exploitable *via* databases, it may be sufficiently complicated to model due to several different statistical/algorithmic approaches to integrate to discriminate significant performance factors. So, instinct or calculation? This is the real question that seems to have to be asked before even engaging in modelling (or even prediction) that could tend towards fiction.
